# DIFFERENCES IN WHITE MATTER MICROSTRUCTURE IN MIDDLE-TO-OLDER AGE ADULTS WITH CHRONIC KNEE PAIN

**DOI:** 10.1093/geroni/igad104.3282

**Published:** 2023-12-21

**Authors:** Kristina Bell, Larissa Strath, Chavier Laffitte Nodarse, Pedro Valdes-Hernandez, Roger Fillingim, Yenisel Cruz-Almeida

**Affiliations:** The University of Florida, Gainesville, Florida, United States; The University of Florida, Gainesville, Florida, United States; The University of Florida, Gainesville, Florida, United States; The University of Florida, Gainesville, Florida, United States; The University of Florida, Gainesville, Florida, United States; The University of Florida, Gainesville, Florida, United States

## Abstract

Abundant research suggests aberrant white matter microstructure in patients with chronic pain as well as in older adults independently, where pain can significantly impact daily function. However, most studies fail to consider pain and aging interactions, particularly in middle and older-age. We aimed to explore differences in white matter microstructure (i.e.,Fractional Anisotropy (FA)) by pain’s impact on daily function (i.e., high (n=60) versus low pain impact (n=111) in middle-(45-59 years old, n=129) versus older adults (60-XX years old, n=77) using diffusion tensor imaging. Participants (n=171) underwent laboratory visits to collect multi-modal pain and neuroimaging measures. We employed a linear mixed model approach to test main effects of pain impact and age groups as well as their interaction with Bonferroni-corrected multiple comparisons. Findings demonstrated a significant pain impact and age interaction on FA (p’s≤0.05) in the left caudal anterior cingulate, left caudal middle frontal, left and right rostral middle frontal and left and right insular regions. Specifically, middle-aged adults with high impact pain had significantly worse brain microstructure across these regions compared to the older adults reporting high impact pain (p’s≤0.05). These findings highlight the need to study pain across the lifespan. Given the higher prevalence of chronic pain in middle-age compared to older age, future studies are urgently needed to test whether interventions in middle-age could halt pain’s impact on brain and function.

